# Intended and suicidal trauma to the anterior neck in Finnish young adults

**DOI:** 10.1007/s00068-025-02813-x

**Published:** 2025-03-20

**Authors:** Riikka E Mäkitie, Silja Kosola, Taru Ilmarinen

**Affiliations:** 1 Department of Otorhinolaryngology– Head and Neck Surgery, Head and Neck Center, Helsinki University Hospital and University of Helsinki, Kasarmikatu 11–13, Helsinki, FI-00029 HUS Finland; 2 Clinicum, Department of Medicine, University of Helsinki, Haartmaninkatu 8, Helsinki, 00014 Finland; 3 Pediatric Research Center, New Children’s Hospital, Helsinki University Hospital and University of Helsinki, Stenbäckinkatu 9, Helsinki, 00290 Finland; 4 Western Uusimaa Wellbeing Services County, Espoo, Finland

**Keywords:** Neck trauma, Intended self-harm, Suicide, Tracheostomy, Neck exploration

## Abstract

**Purpose:**

Self-inflicted injuries are a leading cause of death in young adults. Trauma to the anterior neck, such as from cutting and hanging, can have serious consequences given the complex anatomy and closeness of critical structures. Considering the recent increase in intended and inter-personal violence, we evaluated the occurrence and clinical characteristics of self-harm neck injuries in young adults.

**Methods:**

We retrospectively reviewed all neck traumas treated at the Helsinki University Hospital in patients aged 18 to 30 years in 2005–2023. Patient records were systematically evaluated for cohort demographics, injury type, clinical characteristics, given treatment, follow-up, and possible psychiatric comorbidities.

**Results:**

In total 169 events were recorded, with an evident increase in the recent years (45% of all in 2020–2023) and particularly in females. Females were younger than males (*p* = 0.010) and their trauma generally milder, often managed in outpatient care (82%). Severe penetrating injuries occurred primarily in males (*p* = 0.005) who were older (*p* = 0.004) and without prior suicidal events (*p* = 0.005). They required surgical interventions and prolonged in-house treatment. Overall, the cohort was characterized by a heavy burden of psychiatric comorbidities (98%) and substance abuse (53%); 78% had other suicidal events. Four patients (2.9%) deceased from a recorded suicide during the study period.

**Conclusions:**

We report an alarming increase in suicidal self-harm especially among young females and severe intended neck traumas in older males without preceding suicidal behavior. Our findings warrant timely preventative actions on an individual and societal level and call for refined guidelines for clinical management.

## Background

Self-inflicted injuries are a leading cause of death worldwide, resulting in 800 000 deaths yearly overall, and the second most common cause of death in young adults [1]. Suicide attempts may present as part of a progressive process preceded by thoughts or actions, or, though less commonly, as single independent incidents [2]. Adverse events, such as abuse and experienced assaults predispose to head and neck injury [3]. Moreover, unprecedented circumstances, such as the Covid-19 pandemic or other social and health care stresses alike, may prompt emotional and behavioral distress especially in the most vulnerable young adults.

Trauma to the anterior neck is relatively rare and usually caused by blunt forces from unintentional injuries. Greater forces and especially penetrating injuries disrupting the laryngotracheal complex and/or surrounding structures, however, can lead to severe and possibly life-threatening consequences. Considering the complex anatomy and close proximity of critical structures, neck trauma may be fatal with a higher mortality rate compared to other anatomic sites [4].

Recently, we and others have reported of a distressing increase in neck trauma related to interpersonal and self-inflicted violence resulting in injuries with severe consequences and demanding more extensive treatment [5, 6]. Due to the rarity of severe adolescent neck trauma, proper guidelines for imaging and management are lacking [7]. Given the increase in violent behavior and the possible devastating consequences of neck trauma, protocols for appropriate management are greatly encouraged. We therefore reviewed incidents in the young adult Finnish population in 2005–2023 to improve knowledge of underlying causes, diagnostics, treatment, and long-term consequences of self-inflicted traumas to the anterior neck.

## Materials and methods

### Study cohort

The cohort comprised all young adults (age 18 to 30 years) treated for intended trauma to the anterior neck at the Helsinki University Hospital (referral area 1.6 million) between 2005 and 2023. The age range was selected to include individuals past teenage but prior to mature adult years. The cohort was retrospectively collected from electronic medical records using ICD-10 codes. To avoid selection bias, we searched for ICD-10 codes related to laryngeal and neck trauma: S11 Neck wound, S11.0 Open wound of larynx and trachea, S11.1 Open wound involving thyroid gland, S11.2 Open wound of pharynx and cervical esophagus, S11.7 Multiple open wounds of neck, S11.8 Open wound of other parts of neck, S12.8 Fracture of other parts of neck, S12.9 Fracture of neck, unspecified, S17 Crushing injury of the neck, and S17.0 Crushing injury of larynx and trachea.

The study was approved and granted with an institutional research permission by the Helsinki University Hospital (46/2022).

### Clinical data

We reviewed patient records for patient sex and age, incident type and characteristics, delay to care, findings on initial clinical examination, treatment modality, need for airway management or other surgical intervention, length of stay, long-term follow-up and mortality. Considering the nature of self-inflicted trauma, possible psychiatric histories were also reviewed. Of note, data does not incorporate prehospital suicide deaths or minor injuries not presenting or referred to the University Hospital emergency department. Patient ethnicity was not recorded but patients were assumed to be representative of the Greater Helsinki area population.

### Statistical analyses

Data are presented as frequencies and continuous measures as means (with standard deviations, SD) or medians (with ranges) depending on data distribution. Normality of data was assessed using Kolmogorov–Smirnov and Shapiro–Wilk methods and visually using histograms. Differences and correlations between parameters and subgroups were assessed using Mann–Whitney *U* test (SPSS Statistics 25; IBM Corporation, Armonk, NY, USA). A *p* value < 0.05 was considered statistically significant.

## Results

### Cohort demographics

**Fig. 1:**
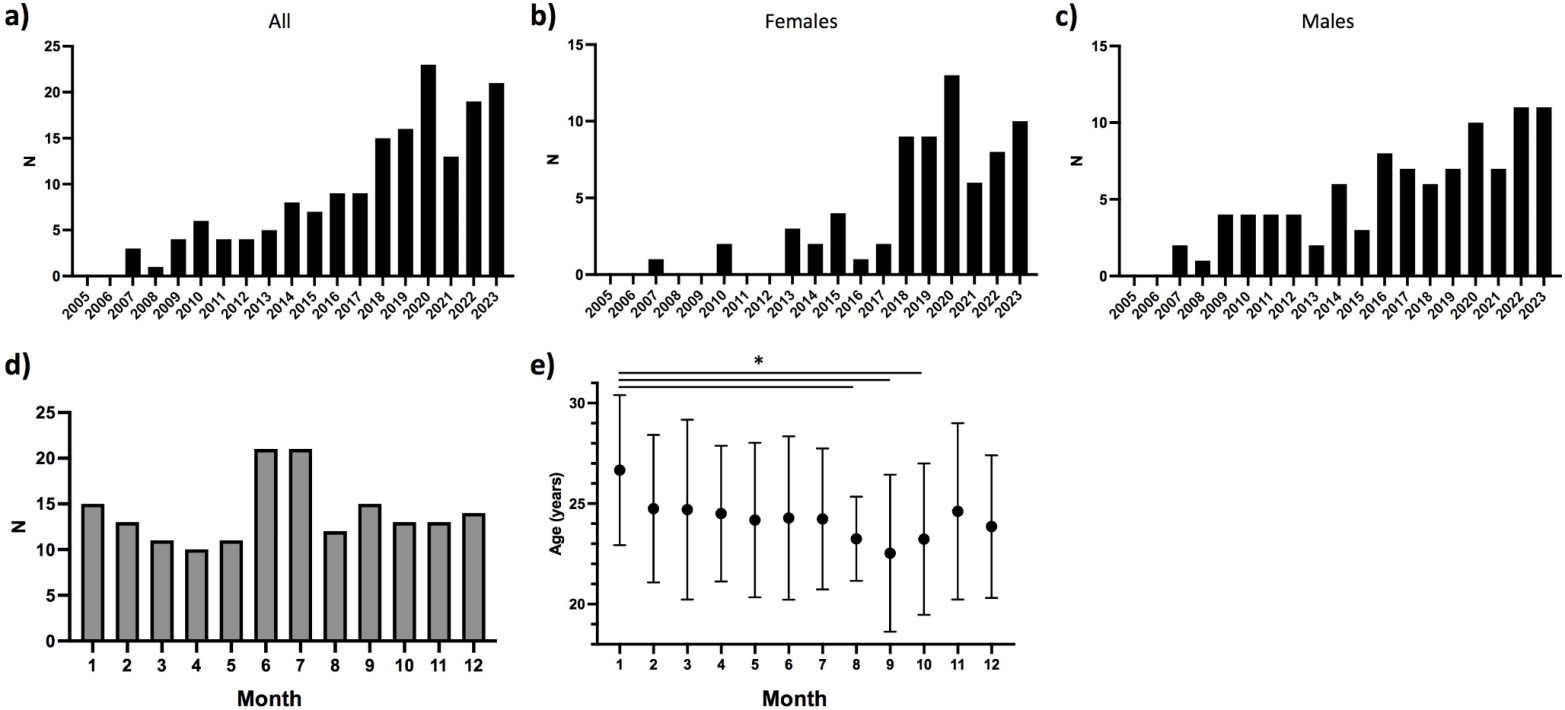
Occurrence of self-inflicted trauma to the anterior neck in Finnish young adults. Figures show an overall increasing trend in the recent years (**a**), which is more pronounce in young females (**b**) than males (**c**). Incidents were more also more common in the summer months (**d**), occurring then in younger patients (**e**). * *p* < 0.05

All patients with a recorded trauma to the anterior neck in result of intentional self-harm were included. Hence the final cohort comprised in total 137 patients (Table 1). Altogether 26 (16%) patients had multiple separate hospital visits (13 males and females) and hence the total number of individual attendances was 167. Occurrence of neck trauma increased in both sexes throughout the study period with 76 cases (46% of all) occurring between 2020 and 2023 (Fig. 1a), though more clearly among females (Fig. 1b and c). Neck trauma were more common and occurred in statistically significantly younger patients during the summer months (Fig. 1d and e). Overall a slight majority of the patients were male (58%). Mean age at first incident was 24.3 years; females were younger than males (mean age 23.3 vs. 24.9 years, range 18–30 for both, *p* = 0.010).

**Table 1:** Cohort demographics and trauma characteristics in Finnish young adults with self-inflicted trauma to the anterior neck

Category	Number (%)
**Patients**	
Total visits	167 (137 individuals)
Males	97 (58.1)
Females	70 (41.9)
Patients with multiple visits	28 (20.4)
**Mean age (years**, **SD)**	24.3, 3.7
Males	24.9, 3.7
Females	23.3, 3.7
**Incident characteristics**	
Walk-ins	15 (9.0)
Brought in by ambulance and/or police	138 (82.6)
Incident at home or other private space	97 (58.0)
Incident in public area	20 (12.0)
Under the influence*	102 (61.2)
**Trauma type**	
Closed	122 (73.1)
Penetrating	45 (26.9)
**Trauma mechanism**	
Cut**	143 (85.6)
Hanging	24 (14.3)
**Psychiatric history**	
Prior psychiatric contact	129 (77.3)
Later psychiatric contact	149 (89.2)
Prior/later suicidal behavior/attempts	131 (78.4)
Alcohol and/or drug abuse	130 (77.8)

* Alcohol and/or drugs

** Cut by knife, razor, glass, or other sharp object

### Incident and trauma characteristics

Most incidents (*N* = 97, 58%) occurred in the home of the patient or their family member, or in similar private environment, 20 (12%) in a public area, 12 (7.2%) at a care facility, 13 (8%) in hospital, and four (2.4%) in a detention facility (Table 1). For 21 cases, data on trauma location was missing. Trauma in a public area were more common among males than females (15 vs. 5).

**Fig. 2:**
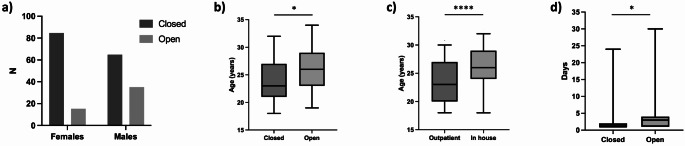
Clinical characteristics and given treatment in self-inflicted trauma to the anterior neck in Finnish young adults. Penetrating/open injuries were more common in males (**a**) and older patients (**b**). Patients admitted to hospital were significantly older (**c**). Length of stay was longer for penetrating/open injuries (**d**). * *p* < 0.05

Most trauma resulted from cuts (143, 86%) or hanging (24, 14%); three patients had both. A knife (typically a kitchen knife) was the most commonly used weapon (82, 57%), followed by a razor blade (11, 7.7%), and scissors and a piece of glass (both 6, 4.2%); in 37 cases (26%) the weapon was not recorded. Majority of injuries were closed or superficial (limited to the skin and subcutaneous fat) (122, 73%) and the remaining 45 (27%) penetrating (i.e. piercing the platysma). Penetrating injuries (34, 76%) were more common in males than females (35% vs. 16%; *p* = 0.005) (Fig. 2a) and older patients (mean age 25.6 vs. 23.7 years; *p* = 0.004) (Fig. 2b). Patients with penetrating injuries were also less likely to have multiple suicidal events compared with patients with closed injuries (66% vs. 86%; *p* = 0.005). Fifty patients (27%) had concurrent trauma in other body locations.

### Clinical treatment

Most patients were brought in by ambulance and/or police (138, 83%), a small portion by nurses from a care facility or ward 11 (6.6%) and 15 (9.0%) came as walk-ins; for three this was not stated. Most patients (157, 94%) were evaluated on the day of trauma (range 0–4 days) and 116 patients (69%) discharged on the same day after initial evaluation and treatment.

Of all patients, 60 (36%) had injuries requiring radiological evaluation (Table 2). Computer tomography (CT) was the most common modality (53, 32%), followed by CT angiography (CTA) (33, 20%), and ultrasound (US) (16, 9.6%). Overall the use of imaging showed an upward trend in the recent years with CT/CTA becoming increasingly common (35 vs. 17%, and 23 vs. 5.7%, for years 2005–2014 vs. 2015–2023, respectively) whereas ultrasound was more commonly applied in the earlier years (7.5 vs. 17%, respectively). Men were more frequently imaged (with any modality) (46% vs. 23%; *p* = 0.002).

**Table 2:** Assessment and treatment of Finnish young adults with self-inflicted trauma to the anterior neck

Category	Number (%)
**Imaging**	
Total	60
Females	17 (23.6)
Males	44 (45.4)
2005–2014	11/36 (28.5) of cases
2015–2023	50/132 (37.3) of cases
**Management**	
Admitted to hospital	51 (30.2)
Treatment in ICU	15 (8.9)
Airway intervention	11 (6.6)
*Intubation*	10 (90.9)
*Tracheostomy*	3 (27.3)
Any surgical intervention	122 (73.0)
*Selective suture/glue*	93 (76.2)
*Surgical exploration*	29 (23.8)
Clinical follow-up	6 (3.6)

Altogether 51 patients (31%) were admitted (Table 2). Inpatients were more commonly male (42% vs. 16%; *p* < 0.001) and significantly older than those treated as outpatients (mean 25.9 vs. 23.4 years, *p* < 0.001) (Fig. 2c). Overall average length of stay in a surgical unit was 3.6 days (range 1–9); this was significantly longer for patients with penetrating injuries than for closed injuries (6.4 vs. 2.5 days, *p* = 0.026) (Fig. 2d). Four patients were hospitalized for 2–4 weeks; they all required care in the ICU, 3/4 needed intubation followed by tracheostomy, and all underwent more extensive surgical treatment. Of all hospitalized patients, 15 (29%) required treatment in the ICU with mean duration of 4.6 days (range 1–8; excluding one outlier of 22 days).

In total 11 patients (6.6%) required airway intervention, either in the form of intubation (10, 91%) and/or tracheostomy (3, 27%) (Table 2). This was typically performed on the day of the event (average 0.2 days, range 0–1) with mean duration of 4.3 days (range 1–8). This excludes two patients for whom decannulation took place on day 18 or 117; a 23-year-old male with bilateral carotid dissection and laryngeal laceration following suicidal hanging, and a 21-year-old female with complete pharyngolaryngeal separation from a deep laceration, respectively.

Overall, 122 patients (73%) needed some form of surgical management (Table 2). For most (93, 76%), this meant wound debridement and suture while 29 (17%) patients underwent surgical exploration and/or suture in the operating room (OR). Surgeries were primarily done on the day of trauma and in four cases on the following day. Patients demanding immediate intervention and surgical exploration had major soft tissue injuries, compromised airway or hemodynamic instability from deep lacerations or extended suffocation. They were predominantly male (72%) and older (26.0 vs. 23.9 years, *p* = 0.004), and comprised 26 (58%) of the penetrating and three (2.5%) of the blunt injuries. These surgeries were often performed by a multidisciplinary team involving otorhinolaryngologists, vascular, cardiothoracic and plastic surgeons.

### Psychiatric morbidity and treatment

Most common psychiatric diagnoses were depression (80, 48%), anxiety (49, 29%), schizophrenia/acute psychosis (39, 23%), and some form of personality disorder (32, 19%). Five patients suffered from PTSD and one from an acute stress reaction. Eleven patients (6.6%) were also on the autism spectrum. Depression was more common in females than males (61% vs. 38%; *p* = 0.003), as was borderline personality disorder (BPD) (39% vs. 5.2%; *p* < 0.001). Of all patients, 102 (61%) presented with a positive toxicology screen, 130 (78%) had documented history of substance abuse, and 90 (54%) had a diagnosed drug addiction. Drug addiction and/or abuse was more common in males (60 vs. 46%; *p* = 0.072). Prior and/or later suicide attempts or suicidal behavior were present in 131 (78%) patient records, more commonly among females than males (89% vs. 74%; *p* = 0.022). Most patients (*N* = 129, 77%) had prior psychiatric contact and this was organized for 149 (89%) after the trauma (including both single evaluation and long-term treatment). Females had more prior (94.0% vs. 66.0%; *p* < 0.001) and later (96% vs. 85%; *p* = 0.022) psychiatric treatments. Interestingly, of patients with penetrating trauma, fewer had prior psychiatric treatment (64% vs. 83%; *p* = 0.012), other suicidal events (66% vs. 86%; *p* = 0.005) or presented under the influence (49% vs. 65%; *p* = 0.068).

### Follow-up and mortality

Clinical follow-up was rare. Clinical follow-up visits for the physical injuries were scheduled for only six patients (3.6%). These were for patients with more severe injuries needing further follow-up. Altogether ten patients (7.3%; 8/10 males) deceased during the study period: one from the recorded trauma, three from a later suicide, and for the remaining six the cause was not stated.

## Discussion

To the best of our knowledge, our study is the first to report findings of intended trauma to the anterior neck in a large Scandinavian young adult population. Our results suggest that the annual occurrence has increased, especially in the recent few years and among young females. Still, most incidents were mild, seldom requiring intensive care or invasive surgical treatment. A high burden of psychiatric comorbidities was observed with many incidents occurring under the influence and in patients with prior and/or later suicidal behavior. These highlight a distressing and growing trend of self-inflicted anterior neck trauma among young adults encouraging improved, targeted interventions and guidelines for management.

Suicide remains the second most common cause of death in the young adult population [8], and in 2022 rates of suicidal idealization and attempts were considerably higher than previously [9]. Recently, an alarming rise in both inter-personal violence and self-inflicted trauma has been observed especially among young adults [10–13]. These events may be mild in nature or associate with more severe trauma done with intention to end life. While adolescents may be less prone to attempt suicidal stab wounds to the neck compared to other body parts, these injuries carry a higher mortality rate of up to 10% [4]. Given the complex anatomy and vital structures, treatment is often demanding and long-term effects potentially traumatizing.

Our review of self-inflicted neck trauma in Finnish young adults found a growing trend and a difference between genders in the recent years. Although events in young females were often mild and treated conservatively in an outpatient manner, such as superficial cuts and scratches or bruising from attempted hanging, reoccurring events happened in a considerable proportion (16%). The more severe and penetrating traumas occurred mostly in males (75.6%), in older patients, and in patients with fewer prior suicidal events. In their review of over 2000 adults with neck trauma, Dodds et al. found that the median age of male patients presenting with penetrating injuries was significantly younger compared to those with blunt injuries (38 vs. 47 years) [14], and others have reported similar gender differences in suicidal behavior and incident rates [15]. Data from Statistics Finland’s database indicate no significant change in actual deaths by suicide in 20- to 29-year-olds but an increase in 15- to 19-year-olds from 2005 to 2023 (increase by 9% and 55%, respectively) [16]. Taken together these support the findings that overall suicidal behavior has increased, and whereas recurrent suicidal behavior is more prevalent in females with actions intended to relieve pain and anxiety, the more severe injuries occur in older males without preceding actions and as single traumatic outbursts with serious intent to end life [17, 18].

Of the 26 penetrating injuries, a large proportion (58%) demanded immediate explorative surgery. This is a higher proportion than reported by others. Stone Jr et al. described a large cohort of 1238 pediatric patients with penetrating neck injuries of whom 24% underwent treatment in the OR, and Tessler et al. reported 10 of their 44 patients (23%) to require neck exploration [19, 20]. The proportion of patients undergoing imaging was also considerably smaller compared to ours (15% vs. 35%). While these differences could be due to variation in patient demographics, trauma types, and study period, institution-based customs and available medical resources are bound to have a role. While guidelines for surgical exploration in adults are more robust, mandated by critical clinical signs and reserved for demanding scenarios, algorithms for managing pediatric and young adult patients with blunt or penetrating neck trauma are less well defined [19–21]. Self-inflicted trauma may be further complicated by severe tissue trauma, systemic physiologic problems and a disturbed mental state possibly in conjunction with intoxication. Surgeries are often performed in a multidisciplinary manner involving several surgical specialties adept at treating and reconstructing complex traumas of the neck. Given the rarity of these trauma, enhancing hospital preparedness by defined guidelines and a named trauma team may enable timely intervention, allocation of resources and preventing further complicating sequalae.

We found a high proportion of lacerations and stab wounds (86%) as well as of psychiatric disorders and substance abuse. A prior large review by Oden et al. reported a high prevalence of mood disorders among patients with nonfatal suicide attempts by self-inflicted stabbings [22]. Other studies have similarly reported that while cuttings are the leading cause of nonfatal self-harm incidents, they represent only 2% of suicides—of which most are typically caused by firearms, suffocation and poisoning [8, 9]. Furthermore, depressive and bipolar disorders have been associated with an increased risk of suicidal behavior and cuttings/piercings to be a common method [23, 24]. Next to this, our finding of depression in only 48% of our patients is interesting. Lastly, and of note, besides self-harm by injurious acts, several studies have reported, likely in consequence to increasing use and availability of drugs and other chemicals, an evident and global increase in adolescent suicidal behavior and near-fatal attempts by overdose as well [25, 26]. While the association between psychiatric comorbidities and suicide risk is evident, many studies are inconsistent in reporting relevant psychiatric information in suicide attempts and thus our current understanding of the major underlying diagnoses and the signs insinuating suicide risk remains incomplete and perhaps therefore inadequately addressed [22]. Considering these increasing trends of self-harm in young adults, expanding knowledge of available support resources for mental health problems on the population level is warranted.

During societal and health care crises, such as the pandemic era, both our acknowledgement and ability to predict and care for shifts in young adults’ psychosocial wellbeing are challenged. Prior studies on the effects of Covid19 on suicidal behavior are scarce and partly conflicting. A large global meta-analysis by Dubé et al. reported a 5 to 10% increase in suicidal and self-harm events and Flesher et al. found a clear increase in self-harm trauma with a high proportion of firearms resulting in more severe (ISS > 6) injuries [6], while Pirkis et al. and Faust et al. found no evident change compared with prior years [27–29]. These discrepancies could be due to differences in evaluated populations, geographical areas and pandemic timepoints, and today the general understanding is that the pandemic period led to an increase in suicidal ideation and attempts while death rates by suicide might have stayed constant [30, 31]. Such changes in incidents could be related to altered psychosocial wellbeing, increased alcohol use and increased gun violence, which may have long predated the pandemic years and only erupted in a disturbed social setting. Noteworthy are also microenvironmental and seasonal changes, such as the increase and shift in patient age during the summer and early autumn months as also reported by for example Holopainen et al. from Finland and Sweden and Nishimura et al. in a Japanese cohort [4, 32]. Thus, environmental factors contributing to psychiatric ill-being and the lead-up to suicide attempts call for concern and improved care. Prior studies have introduced various prevention services, such as school- and community-based programs, interventional frameworks, and improved allocation of public health resources to tackle mental health problems specifically in the young [33, 34]. As such, emergency departments and caring healthcare professionals have a key role often serving as a primary contact for persons at risk.

We recognize some limitations to our study, namely regarding the retrospective study setting and relatively small cohort size. As clinical data were recorded throughout several years and by multiple medical professionals, data are partly incomplete and variable. Furthermore, during the early years of our study period, the number of community level medical emergency centers was higher, and our data also excludes cases of prehospital suicide deaths as well as more minor intentional self-inflicted trauma not warranting clinical evaluation, possibly introducing bias and obscuring true numbers. Increased awareness and enhanced reporting of violence-related injuries might also have introduced bias and contributed to the higher prevalence in the recent years. The reported suicide attempts by means of neck trauma should also be evaluated against self-harm incidents by other means, such as caustic ingestion and poisons, actual deaths by suicide from cutting or hanging, and perhaps on a national platform. Furthermore, expansion of the age criterion to include also older adults might have provided valuable insight into the differences in prevalence, severity and trauma mechanisms by specific age groups—subject for future analyses. Despite, self-harm behavior is commonly underreported, and the young adult population often overlooked in the context of adult cohorts. We therefore consider our findings to provide comprehensive data on neck trauma from a 20-year period and to provide important and novel information on the current status of intentional self-harm among young adults.

In conclusion, we report of a troubling rise in self-inflicted neck trauma and suicidal behavior in young adults in the recent years. The increase in trauma was seen among both genders, and although most trauma were generally mild, some occurred recurrently and with a heavy burden of mental health disorders. Older young adult males seem to constitute a distinct, vulnerable group with more severe and extensive trauma needing surgical and prolonged management and often presenting without prior history of mental health problems or anticipated suicidal behavior. Guidelines for clinical management of severe and complicated trauma, though less common, should be defined and multidisciplinary trauma teams trained to allow timely intervention, proper allocation of resources and hospital preparedness to ultimately prevent delayed care and life-threatening sequalae. Continued surveillance and improved strategies to support the young, whether in the time of crisis or stability, are strongly encouraged.

## Data Availability

Data is provided within the manuscript.
